# Fentanyl or Morphine? a qualitative investigation of solo responding paramedics´ decision-making in prehospital care

**DOI:** 10.1186/s13049-026-01608-2

**Published:** 2026-04-15

**Authors:** Andreas Gustavsen, Randi Simensen

**Affiliations:** 1 Stavanger University, Stavanger, Norway; 2 Division of Prehospital Services, Oslo University Hospital, Oslo, Norway; 3 Institute of Clinical Medicine, University of Oslo, Oslo, Norway; 4 Department of Research and Development, Norwegian Air Ambulance Foundation, Oslo, Norway; 5 Prehospital Division, Innlandet Hospital Trust, Moelv, Norway

**Keywords:** Prehospital care, Morphine, Fentanyl, Decision-making, Solo responding unit, Paramedics, Qualitative, Pain, Opioids

## Abstract

**Background:**

Solo responding unit (SRU) paramedics at Oslo University Hospital (OUH) can administer either morphine or fentanyl for prehospital pain management. Although both opioids are available, the choice between them is made by individual clinicians in time-critical situations, often with limited information, variable transport conditions, and minimal organisational feedback. Existing research has primarily focused on comparative efficacy and safety, while less attention has been paid to how paramedics reason in practice. This study explores the clinical, logistical, and organisational factors that influence SRU paramedics’ opioid selection, how advantages and disadvantages of morphine and fentanyl are perceived, and how local norms and decision-making support shape practice.

**Methods:**

This qualitative descriptive study employed three face-to-face focus groups with a total of 11 participants. The sessions were recorded, transcribed, and analysed thematically. The first author’s dual role as an SRU paramedic was acknowledged and used reflexively to enhance the interpretation of the findings.

**Results:**

The participants (1 female, 10 male, mean age 42 years, mean 19.6 years of ambulance experience from urban and rural regions within OUH) emphasised the role of personal experience and intuitive judgement in selecting between morphine and fentanyl. Fentanyl was favoured for rapid onset in acute traumatic pain or short transports, while morphine was selected for its longer duration in frail patients or lengthy transports. “Ambulance truths”, informal, station-specific beliefs, filled gaps where formal guidance was lacking. Safety concerns existed for both drugs, although severe adverse events were rarely experienced by participants.

**Conclusion:**

SRU paramedics’ opioid selection is shaped by an interplay of pharmacological reasoning, familiarity, organisational protocols, and cultural norms. The findings suggest that formalised training, streamlined documentation, and structured feedback mechanisms may support more consistent decision-making in prehospital analgesia.

**Supplementary Information:**

The online version contains supplementary material available at 10.1186/s13049-026-01608-2.

## Introduction

Effective prehospital pain management is essential to improve patient comfort, reduce physiological stress, and facilitate optimal conditions for definitive care upon arrival at the hospital [1, 2]. For paramedics operating as solo responders, clinical decisions, including the choice of analgesics, must be made independently, often under conditions characterised by limited information, high urgency, and significant clinical uncertainty [3].

In the prehospital services provided by Oslo University Hospital (OUH), solo responding units (SRUs) constitute a specialised resource aimed at rapidly responding to critically ill or injured patients. Unlike regular ambulance crews, SRU paramedics operate alone and are responsible for initial assessment, advanced clinical interventions, and critical decision-making prior to the arrival of supporting resources, often in resource-constrained environments where rapid and efficient decision-making is essential. Their decision-making is highly individualised. Fentanyl was introduced into the SRU formulary in 2022 as a top-down organisational decision by Oslo University Hospital. The implementation was supported by mandatory e-learning modules and scenario-based training, ensuring competence before use. Fentanyl was intended as a supplement rather than a replacement for morphine, giving paramedics two opioid options depending on clinical judgement. Despite the availability of both opioids, limited understanding remains of how paramedics choose between these drugs in various clinical and operational contexts, although the pharmacokinetics and pharmacodynamics of the two drugs are different (Table 1).

**Table 1:** Pharmacological comparison of Fentanyl and Morphine available to SRU paramedics

Feature	Fentanyl	Morphine
Potency^a^	10 µg	1 mg
Onset of Action	1–2 min intravenous (IV)	5–10 min IV
Duration of Action	30–60 min IV	3–4 h IV
Lipid Solubility	High (crosses blood–brain barrier quickly)	Lower
Administration Routes^b^	IV	IV, intramuscular
Side Effects	Respiratory depression, sedation, nausea, constipation, muscle rigidity (high doses)	Respiratory depression, sedation, nausea, constipation, histamine release (causing hypotension and itching)

[4, 5]

^a^Potency refers to the relative pharmacological strength of the drug, not the typical doses administered. Fentanyl is routinely diluted to enable careful titration

^b^Available to SRU paramedics at OUH

Existing studies comparing morphine and fentanyl in prehospital care have mainly focused on measurable outcomes, such as reductions in pain intensity on the numeric rating scale (NRS) and the occurrence of adverse events [6–8]. Although such studies provide essential data on clinical efficacy and safety, they do not address how paramedics reason in real-time or how their decisions are influenced by nonpharmacological factors such as individual experience, organisational culture, or logistical considerations.

Decision-making in prehospital emergency medicine frequently involves navigating clinical uncertainty, constrained resources, and the competing demands of urgency versus safety. Paramedics’ clinical choices may therefore not solely reflect formal protocols or clinical guidelines, but also their professional experience, intuitive judgement, and informal norms established within their working environment [3, 9, 10]. Previous studies have highlighted the need for deeper qualitative insights into the cognitive processes underpinning paramedics’ clinical decisions, noting that a lack of such understanding may lead to variability in clinical practice and potentially suboptimal patient outcomes [1, 3].

Furthermore, paramedics’ decisions regarding analgesic administration may reflect how they perceive risks versus benefits in specific clinical scenarios. For example, fentanyl’s rapid analgesic onset may appear highly advantageous in short-duration or acute-trauma cases, whereas morphine might be perceived as preferable due to its longer analgesic effect during prolonged transports or complex patient presentations [11, 12]. However, perceptions of medication risk, such as fentanyl’s potency-related concerns versus morphine’s predictable yet more frequently observed side effects, may significantly influence choices, especially in the absence of clear organisational guidance or feedback mechanisms.

Organisational factors, including institutional protocols, administrative procedures, and professional culture, also play a critical role in shaping paramedic practice [13–16].

Currently, qualitative research examining these contextual and cognitive aspects of opioid selection in prehospital pain management remains limited [1, 3]. Consequently, this study aims to explore the clinical, logistical and organisational factors influencing SRU paramedics’ decisions between morphine and fentanyl in prehospital settings. Enhanced understanding of these decision-making processes is essential to inform targeted educational strategies, refine clinical guidelines, and ultimately improve patient outcomes and operational consistency within prehospital emergency services.

By applying decision-making, pain management, and organisational behaviour theories [2, 11, 13, 14, 17, 18] the study seeks to uncover the underlying considerations that guide paramedics’ opioid selection, including pharmacological knowledge, logistical constraints, and institutional norms.

This has been operationalised in the following research questions:

1. What are the key factors influencing SRU paramedics’ choice of analgesia in prehospital settings?
2. How do paramedics perceive the advantages and disadvantages of morphine versus fentanyl in the context of SRU operations?
3. To what extent do organisational norms and logistical constraints shape opioid selection in SRUs?
4. What strategies for improving decision-making support and knowledge-sharing regarding prehospital opioid administration could be implemented?

## Methods

### Study design and methodological approach

Focus group interviews were selected as the most appropriate data collection method to explore how solo-responding paramedics collectively make sense of analgesic decision-making within a shared professional context. Focus groups are well suited to examining interaction, negotiation of meaning, and tacit professional knowledge, as discussion among peers can elicit perspectives that may not emerge in individual interviews as described in qualitative methodology literature [19, 20]. Given that prehospital decision-making is embedded in organisational routines and shared experiential knowledge, this method was considered appropriate to capture both convergent and divergent understandings. The approach therefore aligned with the exploratory and interpretive aims of the study.

We conducted a qualitative descriptive study using focus groups, analysed thematically,

[21, 22] and reported according to the COREQ (Consolidated Criteria for Reporting Qualitative Research) checklist (see Supplementary File 1).

Three focus groups were organised in January 2025:

1. Focus Group 1: Central location, mixed urban participants, with 4 attendees.
2. Focus Group 2: Also central, urban and rural participation, 4 attendees.
3. Focus Group 3: Held at an ambulance station in the northern district to encourage rural participation; 3 attendees.

The participants (*n* = 11) had a mean age of 41.9 years (range 31–53). They had on average 19.6 years of ambulance experience (range 12–32) and 5.8 years of Single Responding Unit experience (range 0.5–12) (Table 2). The average duration of focus groups was 105 min (range 90–115 min).

**Table 2:** Participant demographics

Age	Unit	Years in Ambulance	Years SRU	Level of Education	% specific SRU Employment
41	Urban	17	8	NP^a^	0
43	Urban	23	12	NP, MSc^b^	0
43	Urban	25	3	NP	50
31	Urban	12	4	NP	0
41	Urban	17	4	NP, RN^c^	50
53	Urban	32	5	NP	50
42	Rural	23	5	NP, RN, MSC	0
48	Rural	18	0.5	NP	50
42	Rural	19	10	NP	50
35	Rural	15	5	NP	50
42.0	Rural	19	10	NP	50

^a^NP—Two-year post-secondary vocational programme (60 ECTS credits)

^b^*MSc* Master´s degree

^c^*RN* Registered Nurse

The focus groups were organised to allow exploration of both shared and context-dependent perspectives on analgesic decision-making among solo-responding paramedics. Groups were composed of participants with similar professional roles to facilitate open discussion of tacit practices and informal norms, while variation in operational context, including urban and rural deployment, was sought across groups to support analytic comparison [19]. Since the thematic collection of experience is relatively new to the SRU paramedics, it was considered that a group setting could lead to more openness, reflection and courage in the discussions than individual interviews [22]. The study aimed to ensure a balanced representation of both urban and rural SRUs in all focus groups, but this proved challenging. Prior to each focus group, heavy snowfall led to last-minute cancellations, particularly among paramedics working in rural areas. To mitigate this, the final focus group was held at a decentralised location to facilitate participation and ensure diverse perspectives were represented. These logistical constraints reduced the number of participants per focus group from the planned 6–7 to 3–4. Despite the reduced participation, data saturation was considered sufficient after the third focus group because of high information richness from the open discussions [22, 23].

### Setting and participants

The prehospital services in southeastern parts of Norway are provided solely by OUH. Catchment is about 1.5 million people and encompasses amongst others 55 regular emergency ambulances, two Helicopter Emergency Medical Services (HEMS), a physician staffed rapid response car and six SRUs operating in different geographical areas, with five units utilising cars and one utilising a motorcycle. SRU paramedics at OUH receive enhanced training compared to standard ambulance paramedics, including advanced pharmacology and solo decision-making responsibilities. All participating paramedics at OUH operated SRUs, which are single-staffed units responding to both urban and rural emergencies. The inclusion criteria required paramedics to be actively working in an SRU, have at least two years of experience, and be authorised to administer fentanyl and morphine. Recruitment was conducted via a Microsoft Teams channel used to coordinate all the SRU paramedics at OUH.

### Data collection

A semi-structured topic guide covered:

- Clinical experiences with morphine vs. fentanyl.
- Organisational guidelines and perceived barriers.
- Patient factors and logistics (e.g., transport times).

Prior to undertaking the first focus group, a pilot with four participants was conducted. The results were not included in the analysis. Afterwards the questions were refined, and several changes were made to the topic guide. Focus group discussions were recorded and transcribed verbatim. Field notes were taken during and immediately after each focus group to capture contextual observations and reflections on group dynamics. The notes were used reflexively to support interpretation of the transcripts, inform analytic discussions during coding, and contextualise emerging themes. Field notes were not treated as separate data, but as an analytic aid to enhance reflexive awareness throughout the analysis. All sessions were transcribed by Gustavsen using Nettskjema recording and transcription tool developed and hosted by the University of Oslo (nettskjema@usit.uio.no). NVIVO 15 was used for coding.

### Data analysis

Data were analysed using a reflexive thematic analysis following the six-phase approach described by Braun and Clarke [24]. All focus group interviews were transcribed verbatim and analysed iteratively using NVivo. Initial coding generated a large number of data-driven codes across the three focus groups, with decreasing numbers of new codes as analysis progressed. During early theme development, several candidate themes were identified; however, overlap between codes and premature theoretical alignment prompted a re-analysis. All initial themes were therefore discarded, and the data were re-examined with a more inductive focus. Through repeated review of transcripts, codes, and reflexive field notes, codes were grouped, refined, and reorganised into coherent patterns. This iterative process resulted in three overarching themes that were grounded in participants’ accounts and consistently represented across the dataset. Themes were refined through ongoing discussion with the supervisor to ensure clarity, coherence, and alignment with the research aims.

All quotes have been translated from Norwegian to English by Gustavsen and cross-checked by the co-author to ensure accuracy and preserve meaning*.*

### Consent and approval

All participants gave written and verbal consent. Identifiable data were removed except for workplace location (urban/rural). The study was approved by the Norwegian Agency for Shared Services in Education and Research (ref. 141,107, 11 Nov 2024); additional ethical approval was not required.

## Results

The number of new codes identified declined progressively, from 122 in the first focus group to 62 in the second and 26 in the third, with no novel insights emerging thereafter. This suggests that thematic saturation was reached after the third focus group. The analysis resulted in three overarching themes: Organisational and social norms, individual decision-making and clinical considerations.

These themes encompassed several analytically related subthemes, including knowledge-sharing gaps and “ambulance truths”, personal experience and intuitive decision-making, differentiation between fentanyl and morphine in practice, organisational protocols and administrative barriers, transport times and logistics, and safety concerns and fear of adverse effects (Table 3). In this study, the term “ambulance truths” is used to describe locally shared, experience-based understandings articulated by participants, reflecting collectively constructed beliefs that emerge in the absence of formal feedback or structured knowledge exchange.

**Table 3:** Summary of Themes and Sub-Themes Emerging from Focus Groups

Theme	Sub-theme	Illustrative quote
**Organisational and social norms**	Knowledge-sharing gaps and “ambulance truths”	“There’s no real channel for us to say ‘This is how fentanyl worked in these cases’. So, we end up building our own little truths.” (Rural paramedic, FG2)
	Organisational guidelines and administrative barriers	“The most important aspect should be patient care. I don’t think the guidelines have the same focus.” (Urban paramedic, FG1)
	Fear-based implementation strategies	“The training was basically a scare campaign. I was afraid of using it and of administering high enough doses.” (Urban paramedic, FG1)
**Clinical considerations**	Differentiating fentanyl and morphine in practice	“I administer fentanyl for cardiac pain… I choose morphine for back pain.” (Urban paramedic, FG1)
	Transport time and logistical constraints	“With morphine, the patient may sleep all the way to the hospital. It’s not the same with fentanyl.” (Rural paramedic, FG3)
	Safety concerns and fear of adverse effects	“Adverse events are more likely to occur with morphine than with fentanyl, especially when you are alone.” (Urban paramedic, FG1)
**Individual decision-making**	Personal experience and intuitive judgement	“Once you get to know the drug, the decision-making goes quicker.” (Urban paramedic, FG1)
	Familiarity and uncertainty	“I ended up reverting to morphine whenever I doubted how to manage fentanyl in the moment.” (Rural paramedic, FG2)

### Theme 1: Organisational and social norms

Across all focus groups, participants described pronounced knowledge-sharing gaps, which contributed to the development of locally constructed “ambulance truths” based primarily on anecdotal experience rather than formal feedback mechanisms. As a paramedic primarily working rurally described it,

*“There’s no real channel for us to say, ‘This is how fentanyl worked in these cases, maybe we can share that?’ So, we end up building our own little truths”* (rural paramedic 1 FG 2).

Paramedics in urban areas often relied on brief handovers between shifts, while those in rural settings more regularly collaborated with other SRUs stationed alongside them. This influenced how quickly new practices, like using fentanyl, spread within each service and creating differences of practice between different geographical stations. But between all groups there was an expressed lack of system willingness to collect the experiences from the paramedics. This was considered an obstacle when trying to improve practice,

*“Since there is no exchange of experiences, you build all your competence on your own calls, and in that way, it takes a lot of time to improve”* (urban paramedic 3 FG 1).

Participants also highlighted organisational guidelines, administrative barriers, and fear-based implementation strategies as influential contextual factors shaping analgesic practice. Several SRU paramedics described fentanyl as “administratively heavier” than morphine, citing additional documentation requirements and clinical assessments that accompanied its use. This added workload, combined with perceived misalignment between protocol and real-world demands, led to a common understanding between the focus groups that the guidelines hindered good patient care. While a few expressed concerns about deviating from established procedures, others emphasised the primacy of patient comfort over rigid rule-following. As one urban-based paramedic stated:

*“If there is a mismatch between guidelines and practice, it doesn’t necessarily mean that practice is wrong”* (Urban Paramedic 2 FG 2).

This sentiment was shared by others who shared a patient-centred focus rather than a guideline-focused:

*“The patient has a right to be relieved from pain, and we shouldn’t expose them to more stress or pain than necessary”* (Urban Paramedic 3 FG 1).

Participants also identified gaps in training and information flow across the prehospital system. While SRU paramedics had received targeted fentanyl training, general ambulance crews responsible for continuing patient care during transport, often had not. One rural paramedic described this disconnect:

*“You provide quick information regarding the patient and the drugs administered, but then you close the ambulance doors, and they drive off. The others* [paramedics in the service] *should also have been trained and informed, since they are the ones who observe the patients during transport to the hospital.”* (Rural Paramedic 2 FG 3).

Another theme that emerged across focus groups was the experience of fear-based messaging during fentanyl implementation. Participants described the training as overly cautionary, and saw this as something they had to overcome to use the drug efficiently:

*“The training was basically a scare campaign. I was afraid of using it* [fentanyl] *and of administering high enough doses”* (urban paramedic 3 FG 1).

*“We were left with an impression that fentanyl almost was too dangerous to administer”* (rural paramedic 1 FG 2).

This characterisation of the implementation process as ‘scare tactics’ was universally shared by participants across all focus groups either by verbal description or by nodding or other signs of agreeing.

### Theme 2: Clinical considerations

Clinical considerations related to the pharmacological differences between fentanyl and morphine were central to opioid selection, particularly regarding onset of action, duration, and anticipated clinical trajectory. The participants’ accounts of the differences between fentanyl and morphine largely aligned with established pharmacological knowledge. Fentanyl was consistently favoured for its rapid onset and short half-life, particularly in the management of acute traumatic pain, such as fractures or severe lacerations. In situations involving short extrication times or the need for rapid patient transfer, fentanyl was described as the more appropriate choice. In contrast, morphine was perceived as advantageous for its longer duration of action, which reduced the need for repeated dosing during prolonged transports or extended handovers at the hospital.

However, in actual clinical practice, opioid selection was often shaped less by textbook pharmacokinetics and more by logistical considerations, such as distance to hospital, anticipated clinical trajectory, and available scope of practice. Several participants noted that concerns about polypharmacy frequently prompted them to commit to a single opioid. In rural areas, where ambulance paramedics could only administer morphine, SRU clinicians often opted for morphine from the outset, anticipating the potential need for redosing en route.

*“We have been instructed to avoid mixing opioids. That means that if they send you, and you start out with fentanyl, you should be really confident that they reach the hospital without the need for pain medicine unless you intend to accompany the patient yourself”* (urban paramedic 5 FG 2).

But this concern was not shared by all. Others saw no issues in mixing, and some were actively taking advantage of the different characteristics of the drugs,

*“If a patient reports a NRS of 10 and is screaming of pain, … I may start administering fentanyl and later switch over to morphine because I expect things to take a long time”* (rural paramedic 1 FG2).

Patient vulnerability was another key consideration. For older or multimorbid patients, many paramedics expressed a greater sense of control when using morphine, citing its well-known side effect profile, even when they acknowledged fentanyl might have offered superior pharmacological benefits.

Paediatric cases generated more divergent views. Some participants preferred fentanyl for its ease of titration and rapid response in children, while others defaulted to morphine based on habit and comfort. Despite these nuances, there was broad agreement across all focus groups: SRU paramedics tended to use fentanyl in situations requiring immediate, high-impact analgesia, and morphine in scenarios involving sustained, complex pain management.

Transport time and logistical constraints were repeatedly described as integral clinical considerations influencing opioid choice, particularly in rural settings with prolonged extrication and transfer.

Urban SRUs appreciated fentanyl’s rapid effect for short transfers. Paramedics on rural SRUs were more inclined to choose morphine due to its prolonged effect and sustained relief.

*“If the patient faces a prolonged extraction and transport, I will probably choose morphine”* (urban paramedic 2 FG 1).

*“Because of the distances in our district, I feel it´s more of a hassle to the patient to titrate and maintain the effect of the drug. If I can reach proper pain relief with morphine, the patient may sleep all the way to the hospital. I don’t think the patients that are given fentanyl get the same good sensation due to the short half-life” (rural paramedic 2 FG 3).*

Concerns about safety and fear of adverse effects further informed clinical reasoning, with participants weighing perceived risks of respiratory depression, hypotension, and sedation when selecting opioids. For some paramedics, morphine was perceived as the safer and more predictable option, especially in vulnerable patient groups. One rural paramedic explained:

*“Really old and multimorbid patients … I would give them morphine even though I don’t think there is a good reason for it”* (rural paramedic 1 FG 2).

Others viewed the risk profile differently, emphasising that morphine carried a higher likelihood of complications, especially in solo practice settings:

*“Adverse events are more likely to occur with morphine than with fentanyl. This is even more important when you are alone.”* (urban paramedic 4 FG 1).

Despite the perceived risks, many participants regarded fears around fentanyl as exaggerated. Respiratory depression was described as rare, and when it did occur, it was typically manageable. While a few noted occasional episodes of sedation or euphoria associated with morphine, these effects were not seen as clinically negative. Several participants also differentiated between the perceived sedative effect of morphine, often felt by the patient, and a more subtle, observed sedative effect from fentanyl, which was primarily noticed by the clinician.

### Theme 3: Individual decision-making

Individual decision-making was strongly shaped by personal experience and intuitive judgement, particularly in time-critical situations where rapid opioid selection was required. While fentanyl was often praised for its rapid onset, it was initially perceived as intimidating by all paramedics following the implementation. Several paramedics described making a deliberate effort to incorporate fentanyl into their practice once it became widely available, noting that repeated clinical exposure gradually increased their confidence. As one urban-based paramedic explained:

*“Once you get to know the drug* [fentanyl]*, the decision-making goes quicker”* (urban paramedic 1 FG1).

In contrast, participants who had less familiarity with fentanyl reported reverting to morphine in moments of uncertainty, relying on what felt more predictable in the immediate situation:

*“I ended up reverting to morphine whenever I doubted how to manage fentanyl in the moment”* (rural paramedic 1 FG2).

Overall, familiarity frequently outweighed theoretical advantages. However, as paramedics accumulated hands-on experience and observed positive patient outcomes, they reported a growing tendency to select fentanyl more instinctively and with greater ease.

## Discussion

This study identified three interrelated themes influencing opioid choice among solo-responding paramedics: organisational and social norms, clinical considerations, and individual decision-making. While fentanyl and morphine were perceived as clinically effective when titrated appropriately, participants described how non-clinical factors such as organisational constraints, informal norms, and personal experience shaped real-time analgesic decisions. Understanding underlying mechanisms behind decision making is important, as the ability to choose the most appropriate analgesic based on situational factors is crucial for optimising patient outcomes. Quantitative studies highlight that fentanyl and morphine offer similar analgesic efficacy if titrated properly [6, 7]. However, our study suggests that “intangible” factors, like comfort, fear of adverse outcomes, and bureaucratic burdens, have a substantial impact on real-time decision-making, alongside clinical guidelines. The discussion is structured around the three overarching themes identified in the analysis, with each section relating the empirical findings to relevant theoretical perspectives and existing literature.

### Integrating decision-making theories

The findings of this study suggest that opioid choice among solo-responding paramedics is influenced by a combination of clinical considerations, organisational context, and individual experience. Participants described assessing pharmacological properties, patient characteristics, and logistical factors, but also emphasised the role of familiarity, uncertainty, and local practice norms in real-time decision-making.

These findings can be interpreted using established decision-making theories. Dual-process theory distinguishes between fast, intuitive decision-making (system 1) and slower, more analytical reasoning (system 2) [17]. In the prehospital setting, where decisions are made under time pressure and with limited information, reliance on intuitive judgement may represent a pragmatic and adaptive strategy. Participants’ descriptions of increasingly rapid opioid selection with growing experience are consistent with this interpretation.

Bounded rationality further highlights how decision-making is constrained by limited information, time pressure, and cognitive load [9]. In this study, paramedics frequently described uncertainty related to follow-up care, re-dosing, and adverse effects, as well as limited opportunities for feedback. These constraints may help explain why morphine was sometimes chosen despite participants recognising fentanyl as pharmacologically advantageous in certain situations.

The theory of planned behaviour provides an additional perspective on how attitudes, social norms, and perceived control may influence opioid choice [13]. Participants described how expectations from colleagues, fear-based implementation strategies, and locally developed “ambulance truths” shaped their practice. Such influences may contribute to discrepancies between what clinicians considered the most appropriate analgesic and the drug they ultimately administered [15].

Together, these theoretical perspectives offer a framework for understanding the variation in decision-making observed in this study. Rather than indicating fixed or causal patterns, they help contextualise how clinical reasoning in prehospital care is shaped by experience, organisational influences, and adaptive responses to uncertainty.

Participants’ accounts suggest that distinctions between fentanyl and morphine in relation to transport time, re-dosing, and downstream care were not consistently articulated as part of formal training or guidelines. Instead, these considerations appeared to develop through experiential learning and local practice. The emergence of locally shared “ambulance truths” illustrates how personal beliefs and perceived peer expectations may shape opioid choice, sometimes contributing to practices that diverge from formal guidelines [13, 18, 25].

### The impact of organisational constraints

Across all focus groups, participants described the documentation and assessment requirements associated with fentanyl administration as extensive and primarily system-oriented rather than patient-focused. While these requirements were often perceived as burdensome, they did not necessarily deter fentanyl use. Several SRU paramedics reported consciously deviating from established protocols when strict adherence was perceived to conflict with patient comfort or clinical priorities.

Digital decision-support tools were also discussed as organisational constraints. The electronic application used for opioid guidance was frequently described as difficult to navigate, leading some participants to bypass it in practice. These accounts suggest a gap between the design of organisational support systems and their usability in time-critical prehospital contexts.

From an analytical perspective, these findings can be interpreted through institutional theory, which distinguishes between regulative, normative, and cognitive mechanisms shaping professional practice [14]. Participants’ descriptions indicate limited influence of formal guidelines (regulative mechanisms), while locally shared norms and peer expectations appeared more influential in shaping everyday practice. The development of locally embedded “ambulance truths” reflects processes of institutional isomorphism, where practices are adapted and stabilised within specific organisational contexts [15].

Cognitive mechanisms, including familiarity and experiential knowledge, further appeared to sustain these locally developed practices. Participants’ accounts of limited feedback and training opportunities suggest that such beliefs may be difficult to modify, highlighting the importance of ongoing education, communication, and organisational learning to support guideline implementation [14].

### Informal knowledge-sharing and its implications

Participants’ descriptions of “ambulance truths” illustrate the dual role of informal knowledge-sharing in prehospital practice. Informal exchanges between colleagues were described as an important source of practical insight and experiential learning, particularly in the absence of structured feedback mechanisms. At the same time, reliance on anecdotal experience may contribute to the persistence of locally embedded practices that are not routinely evaluated against formal guidelines or emerging evidence [26, 27].

This tension between experiential knowledge and formal regulation reflects a broader challenge in prehospital care, where clinical practice is often shaped by situational demands and limited opportunities for systematic reflection. Participants’ accounts suggest that, in the absence of regular debriefing or outcome feedback, informal norms may become stabilised over time, reinforcing both effective and potentially suboptimal practices.

### Implications for training and policy

The findings of this study have implications for both clinical practice and organisational policy. Participants’ accounts suggest that fear-based messaging during the implementation and training of fentanyl may have contributed to prolonged hesitancy in its use. Addressing how new analgesics are introduced, including the balance between risk communication and practical guidance, may support greater confidence and alignment between clinicians’ assessments of the most appropriate drug and their actual practice.

Administrative and documentation requirements were also described as influencing opioid choice. Participants’ reports of routine deviations from guidelines raise questions about whether such practices primarily reflect individual behaviour or limitations in the guidelines themselves. These findings suggest that guideline revision, informed by clinical experience, may be more effective than increased enforcement alone.

Finally, the study highlights the potential value of clinical governance structures that acknowledge and integrate informal experiential knowledge. Participants described “ambulance truths” as an important source of practical learning but also recognised the limitations of relying on anecdotal experience in the absence of structured feedback. Approaches that combine informal knowledge-sharing with regular feedback and evidence-informed reflection may support continuous improvement in prehospital pain management [27]. Organisational change is more likely to be successful when clinicians are actively involved in development processes and perceive the relevance of proposed changes to their everyday practice [28].

### Strengths and limitations

Strengths include the rich discussion enabled by focus groups and the first author’s insider knowledge, which helped elicit deeper insights. Limitations relate to the modest sample size (*n* = 11) and the fact that not all geographical areas were represented. Nevertheless, participants from both urban and rural SRUs contributed, and the findings are consistent with international evidence on paramedic decision-making [3, 25, 29], suggesting that they may be transferable to comparable prehospital contexts, though caution is warranted beyond OUH. The first author’s dual role could have introduced bias; however, reflexivity was employed to mitigate this. As the first author had similar seniority and experience as the participants, power imbalances were unlikely to have influenced the discussions, and guidance from the co-author prior to the focus groups further supported reflexivity.

## Conclusion

This study demonstrates that SRU paramedics’ choice between morphine and fentanyl is influenced by a mixture of pharmacological understanding, personal familiarity, organisational protocols, and cultural norms. Participants described how perceived simplicity, transport considerations, and administrative demands shaped opioid selection in everyday practice. While this study does not assess comparative analgesic efficacy, the findings indicate that organisational approaches such as clearer protocols, structured feedback, and targeted training may support more consistent and confident decision-making in prehospital pain management.

## Supplementary Information

Additional file 1.

## Data Availability

The datasets used and/or analysed during the current study are available from the corresponding author on reasonable request.

## Abbreviations

EMT: Emergency Medical Technician
HEMS: Helicopter Emergency Medical Services
IV: Intravenous
NEWS-II: National Early Warning Score II
OUH: Oslo University Hospital
REC: Regional Committees for Medical and Health Research Ethics
SIKT: Norwegian Agency for Shared Services in Education and Research
SOP: Standard Operating Procedures
SRU: Solo Responding Unit
