# Supplementary material for: Fentanyl or Morphine? a qualitative investigation of solo responding paramedics´ decision-making in prehospital care
Source: Scand J Trauma Resusc Emerg Med. 2026 Apr 15;34:80. doi: 10.1186/s13049-026-01608-2 (PMC13109881; doi:10.1186/s13049-026-01608-2)
Supplement: Supplementary file 1 — Additional file 1. [file 13049_2026_1608_MOESM1_ESM.docx]

COREQ (COnsolidated criteria for REporting Qualitative research) Checklist

| Topic | Item no. | Guide questions/description | Reported on page no. |
| --- | --- | --- | --- |
| Domain 1: Research team and reflexivity |  |  |  |
| Personal Characteristics |  |  |  |
| Interviewer/facilitator | 1 | Which author/s conducted the interview or focus group? | p. 15 |
| Credentials | 2 | What were the researcher’s credentials? E.g. PhD, MD | p. 1 |
| Occupation | 3 | What was their occupation at the time of the study? | p. 15 |
| Gender | 4 | Was the researcher male or female? | p. 15 |
| Experience and training | 5 | What experience or training did the researcher have? | p. 15 |
| Relationship with participants |  |  |  |
| Relationship established | 6 | Was a relationship established prior to study commencement? | p. 15 |
| Participant knowledge of the interviewer | 7 | What did the participants know about the researcher? | p. 15 |
| Interviewer characteristics | 8 | What characteristics were reported about the interviewer/facilitator? | p. 15 |
| Domain 2: Study design |  |  |  |
| Theoretical framework |  |  |  |
| Methodological orientation and theory | 9 | What methodological orientation was stated to underpin the study? | p. 14 |
| Participant selection |  |  |  |
| Sampling | 10 | How were participants selected? | p. 13 |
| Method of approach | 11 | How were participants approached? | p. 13 |
| Sample size | 12 | How many participants were in the study? | p. 16 |
| Non-participation | 13 | How many people refused to participate or dropped out? Reasons? | N/A |
| Setting |  |  |  |
| Setting of data collection | 14 | Where was the data collected? | p. 16 |
| Presence of non-participants | 15 | Was anyone else present besides the participants and researchers? | N/A |
| Description of sample | 16 | What are the important characteristics of the sample? | p. 16 |
| Data collection |  |  |  |
| Interview guide | 17 | Were questions, prompts, guides provided by the authors? Was it pilot tested? | p. 14 |
| Repeat interviews | 18 | Were repeat interviews carried out? If yes, how many? | N/A |
| Audio/visual recording | 19 | Did the research use audio or visual recording to collect the data? | p. 14 |
| Field notes | 20 | Were field notes made during and/or after the interview or focus group? | p. 14 |
| Duration | 21 | What was the duration of the interviews or focus group? | N/A |
| Data saturation | 22 | Was data saturation discussed? | p. 14, 17 |
| Transcripts returned | 23 | Were transcripts returned to participants for comment and/or correction? | N/A |
| Domain 3: Analysis and findings |  |  |  |
| Data analysis |  |  |  |
| Number of data coders | 24 | How many data coders coded the data? | p. 14 |
| Description of the coding tree | 25 | Did authors provide a description of the coding tree? | p. 14, 32 |
| Derivation of themes | 26 | Were themes identified in advance or derived from the data? | p. 14 |
| Software | 27 | What software, if applicable, was used to manage the data? | N/A |
| Participant checking | 28 | Did participants provide feedback on the findings? | N/A |
| Reporting |  |  |  |
| Quotations presented | 29 | Were participant quotations presented to illustrate the themes/findings? Was each quotation identified? | p. 17–28 |
| Data and findings consistent | 30 | Was there consistency between the data presented and the findings? | Yes – throughout |
| Clarity of major themes | 31 | Were major themes clearly presented in the findings? | p. 17–28 |
| Clarity of minor themes | 32 | Is there a description of diverse cases or discussion of minor themes? | p. 27–28 |
